# Influence of Sales Promotion Techniques on Consumers’ Purchasing Decisions at Community Pharmacies

**DOI:** 10.3390/pharmacy7040150

**Published:** 2019-11-08

**Authors:** Younes Ben Said, Nicola Luigi Bragazzi, Natalia Valeryevna Pyatigorskaya

**Affiliations:** 1 Department of Industrial Pharmacy, Sechenov First Moscow State Medical University (Sechenov University), Moscow 119991, Russia; younisbensaid@gmail.com (Y.B.S.); osipova-mma@list.ru (N.V.P.); 2 Postgraduate School of Public Health, Department of Health Sciences (DISSAL), University of Genoa, 16132 Genoa, Italy; 3 Department of Mathematics and Statistics, Laboratory for Industrial and Applied Mathematics (LIAM), York University, Toronto, ON M3J 1P3, Canada

**Keywords:** pharmaceutical sales promotion, consumer’s purchasing decision, prevalence and effectiveness of marketing techniques

## Abstract

This research aims to identify the most prevalent and impactful sales promotion tools used by pharmaceutical companies on consumers’ purchasing decisions at community pharmacies. A cross-sectional study design was carried out using the non-repeated random sampling technique. Standardized questionnaires were administered by means of face-to-face interviews or via emails. The relative importance of prevalence (RIP) and the mean evaluation of effectiveness (MEE) were determined for all studied marketing tools for the different groups of respondents (pharmaceutical sales representatives (PSRs), community pharmacists, consumers, and the entire sample). Inter-individual differences in RIP and MEE were assessed by computing the coefficient of variation, whereas inter-group differences were determined by one-way analysis of variance (ANOVA) with the Scheffé test as a post-hoc test. Research findings showed that, according to all respondents, the consumer promotion technique had the strongest impact on consumers’ purchasing decisions while merchandising was the most common sales promotion technique at community pharmacies. PSRs and pharmacists identified trade promotion as the most effective and prevalent technique. Furthermore, research findings showed that, according to all respondents, the following sales promotion tools had the strongest impact on consumers’ purchasing decisions: arrangement and design of showcases among the studied tools for merchandising; buy 1 and get 2 among the studied tools for consumer promotion; and gifts among the trade promotion studied tools. The same tools were identified as the most prevalent by all respondents. Free samples of promoted products appeared to be the most prevalent tool, but at the same time was the least effective. In conclusion, the results of the present research enable an understanding of which sales promotion tools are commonly used at community pharmacies and which ones have the strongest impact on consumers’ purchasing decisions.

## 1. Introduction 

The pharmaceutical market of the Kingdom of Saudi Arabia (KSA) is one of the largest in the Middle East. It is highly developed and characterized by a wide range of products. It was valued at $5209.5 billion in 2016 and is expected to expand at a compound annual growth rate (CAGR) of 9.0% over the period 2016–2026 [1]. Due to the fact that the dynamic expansion of the pharmaceutical market of the KSA is still ongoing [2], an increasing level of trade competition is observed. This explains why most pharmaceutical companies invest time and money in the field of marketing and try to find more effective promotion tools to further increase their sales and revenues [3,4,5].

Nowadays, the pharmaceutical industry uses a range of promotion techniques at the retail level of the pharmaceutical market [6] to try and reach consumers indirectly by below the line (btl) marketing techniques that stimulate sales [5,7].

The marketing techniques of sales promotion can be divided into three main groups depending on the focus of their impact: (1) consumer promotion (stimulating consumer demand), which represents the implementation of the “pull” promotion strategy of a pharmaceutical company; (2) trade promotion (motivating pharmacists’ trading activity), which implements the “push” promotion strategy [8]; and (3) merchandising (visual demonstration of goods and management of retail space).

The technique of consumer promotion is aimed to increase sales and allow the company to “pull” the buyer. This technique includes both non-price incentive tools (gifts for purchasing the promoted product, free samples, etc.), and price incentive tools (discounts, discount/bonus accumulative programs, offers buy 1 and get 2, etc.) [9,10].

The “push” technique of motivating pharmacists’ trading activity (trade promotion) is intended to drive a product through marketing channels to the consumer [8,11]. It employs different tools designed to motivate the pharmacist to dispense the promoted product to consumers: trade stimulating programs (the pharmacist receives gifts when a certain level of either retail sales or wholesale purchases of the promoted product is reached); btl events such as the secret buyer; free drug samples; etc. [10,12]. Pharmaceutical companies try to build the pharmacists’ loyalty toward the brand [12,13] by organizing events designed to enhance the professional knowledge of pharmacists (scientific conferences, seminars, lectures, etc.) and various training activities (workshops, master classes, etc.) aimed to deepen the active sales skills of community pharmacists [12,14].

The essence of merchandising is to build effective marketing communication between a product and consumers. Merchandising aims to increase the volume of sales. It is always customer oriented, and, according to its principles, everything in the pharmacy should be in sight, accessible, attractive, and convenient for the customer. Merchandising includes a set of tools that create the unique atmosphere of the pharmacy by using light, sound, and color effects; showcase design; the special positioning of showcases, products, and advertising materials, etc. This technique involves using point of sales (POS)-materials and determines their most effective location in the pharmacy. POS-materials serve to attract the consumers’ attention to the products and thereby is more effective. At community pharmacies, POS-materials are presented through various channels such as posters, flyers, shelf-talkers, dispensers, stickers, wobblers, etc. [15]. 

To the best of our knowledge, no previous research has been planned and implemented to identify the most prevalent sales techniques stimulating marketing tools at the retail pharmaceutical markets of the KSA, nor to determine which ones among them most effectively influence consumers’ purchasing decisions. 

## 2. Methodology

The objectives of this research were as follows: to identify the prevalence of tools for the sales promotion techniques used in community pharmacies; and, to determine the most effective sales promotion tools that impact the most on the consumer’s purchasing decision.

### 2.1. Study Design and Sample

A cross-sectional study design was carried out in the community pharmacies using the non-repeated random sampling technique. To obtain statistically reliable results, the sample included the following: 340 community pharmacists, 50 pharmaceutical sales representatives (PSRs), and 400 pharmacy consumers. The socio-demographic characteristics of the sample are presented in Table 1.

### 2.2. The Questionnaire

After a literature review on the topic under scrutiny, a data collection tool (a questionnaire) was developed ad hoc by the authors. The questionnaire was designed and specifically adapted based on the group of respondents (PSRs, community pharmacists, and pharmacy consumers). The questionnaire consisted of two question subsets. The first part included items formulated to explore the prevalence of marketing tools. Respondents were asked to choose the tools used in the community pharmacies from a proposed list. The questions of the second subset were formulated to estimate the effectiveness of the studied marketing techniques. Respondents were asked to evaluate each of the proposed marketing tools according to the strength of the impact on consumer purchasing decisions.

The questionnaires also contained socio-demographic questions. Face and content validity of the questionnaire were assessed by a group of experts from the Sechenov First Moscow State University, Moscow, Russia. Data were collected by means of questionnaires administered via face-to-face interviews in community pharmacies in Riyadh or via mail through Sphinx online software.

### 2.3. Statistical Analysis

Data obtained from the survey were coded and analyzed using the “Statistical Package for Social Sciences” (SPSS for Windows, version 24.0, IBM, Armonk, NY, USA). 

The relative importance of prevalence (RIP) and the mean evaluation of effectiveness (MEE) for each marketing tool were determined for each different group of respondents (PSRs, pharmacists, consumer, and the entire sample). Based on the results obtained, sales promotion techniques and their tools were ranked for prevalence and effectiveness. 

Inter-individual differences in terms of RIP and MEE were assessed by computing the coefficient of variation, whereas inter-group differences were determined by the one-way analysis of variance (ANOVA) and the Scheffé test as the post-hoc test. A *p*-value < 0.05 was considered statistically significant. 

## 3. Results

The determined values of RIP and MEE allowed us to rank all of the studied techniques (in the case of trade promotion technique, the opinions of consumers were not studied due to the fact that consumers were not faced with its implementation) (Table 2 and Table 3). 

### 3.1. Merchandising

Among the numerous merchandising tools, the following were analyzed in the study: POS-materials; arrangement and design of showcases; arrangement of advertising materials; product-magnets; special arrangements of goods; light, sound, and aroma effects.

**Figure 1:**
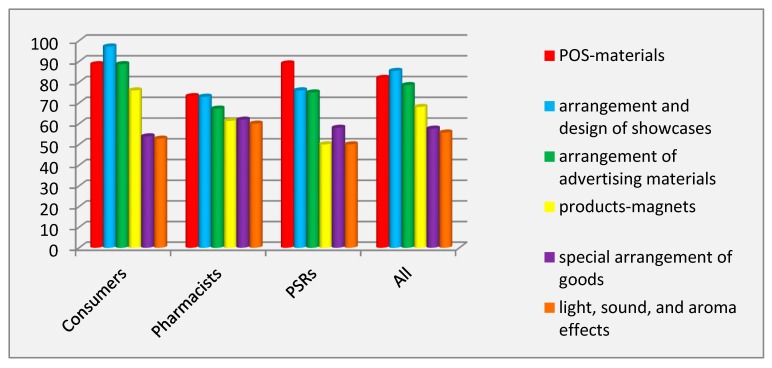
Prevalence of the tools of merchandising. Abbreviations: PSRs (pharmaceutical sales representatives).

The study of the prevalence of merchandising tools revealed that the specific arrangement and design of showcases was considered to be the most common, according to all respondents (RIP = 85.38%). POS-materials ranked second, according to all respondents (RIP = 82.02%) and to the group of pharmacy consumers (RIP = 88.63%), with PSRs (RIP = 89%) and pharmacists (RIP = 73.24%) considering this tool to be the most widespread among the tools of merchandising. The least prevalent, according to all respondents (RIP = 55.70%), was the tool using light, sound, and aroma effects. PSRs employed neither light, sound, aroma effects, nor product-magnets (Figure 1). The values of the coefficient of variation indicated the complete absence of inter-individual differences in the PSRs group (V = 0%) for the named two tools, confirming that these tools were not used at all. In all other cases, inter-individual differences were found. 

All tools of this technique were shown to have a significant effect (*p* < 0.001) of the factor of the respondents’ category on the variation of prevalence from ANOVA analysis. The degree of influence varied from ɳ^2^ = 12.50% to ɳ^2^ = 27.45%. The Scheffé test revealed significant differences between groups of pharmacy consumers and pharmacists for all tools of this technique (*p* ˂ 0.001).

**Figure 2:**
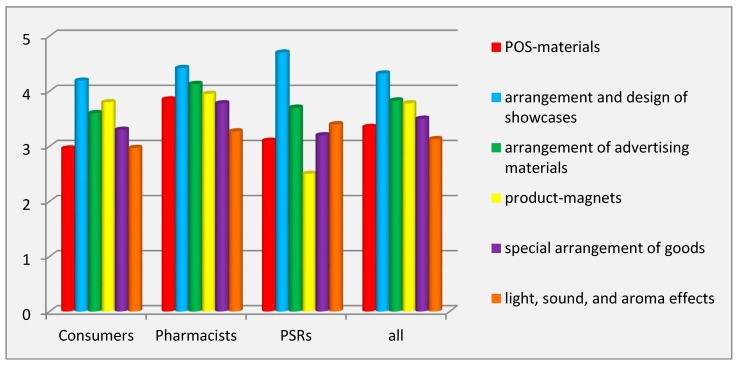
Effectiveness of merchandising tools. Abbreviations: PSRs (pharmaceutical sales representatives).

The study of the effectiveness of merchandising tools showed that, according to all respondents (MEE = 4.32 ± 1.64 points), the specific arrangement and design of showcases had the greatest impact on consumers’ purchasing decision. Similar results were obtained in all other groups of respondents: PSRs (4.70 ± 1.57 points), pharmacists (4.42 ± 1.51 points), and pharmacy consumers (4.19 ± 1.73 points) (Figure 2).

According to all respondents (3.13 ± 1.80 points), the least effective tool of merchandising was using light, sound, and aroma effects. Regarding the least effective tool, the opinions of the pharmacists and pharmacy consumers coincided (3.27 ± 1.74 points and 2.97 ± 1.86 points, respectively), and PSRs considered using product–magnets as the least effective (2.50 ± 0.93 point) (Figure 2). The values of the coefficient of variation (34.14–64.19%) indicated the presence of inter-individual differences in the evaluation of effectiveness of the studied tools.

ANOVA analysis showed significant inter-group differences (*p* ˂ 0.001) for the factor of respondents’ category on the variation of the evaluation of effectiveness, except for the POS-materials (ɳ^2^ = 6.4%, *p* ˂ 0.001). The Scheffé test revealed significant inter-group differences for the effectiveness of the tool product-magnets between PSRs and consumers (1.30 points) and between PSRs and pharmacists (1.45 points) from one another (*p* ˂ 0.001).

### 3.2. Consumer Promotion Technique

Among the tools for consumer promotion, the following were analyzed in the study: discounts; discount accumulative cards; bonus accumulative cards; promoted product and gift; buy 1 and get 2; and free samples.

**Figure 3:**
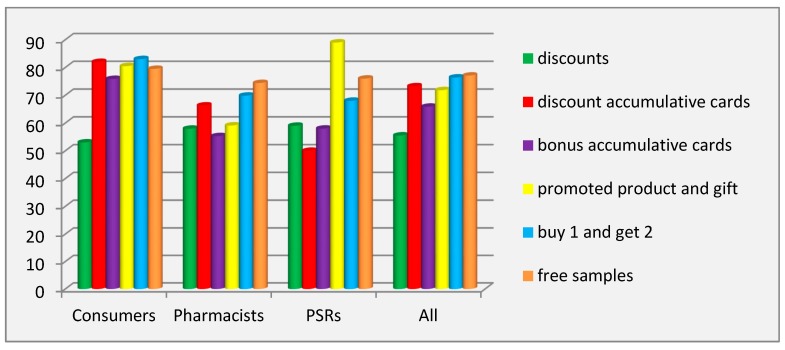
Prevalence of the tools for consumer promotion. Abbreviations: PSRs (pharmaceutical sales representatives).

Findings showed that the most prevalent tools for consumer promotion were free samples of the promoted product (RIP = 77.09%) and buy 1 and get 2 (RIP = 76.39%), according to all respondents. Community pharmacists considered free samples of the promoted product (RIP = 74.41%) as the most prevalent; consumers preferred buy 1 and get 2 (RIP = 83%); and PSRs named promoted product and gift (RIP = 89%) as the most prevalent one. The least prevalent tool (RIP = 55.51%) was discounts, according to all (Figure 3).

The coefficient of variation showed significant inter-individual differences in all groups of respondents for all tools of this technique, except for the tool discount accumulative cards (V = 0%) in the group of the PSRs. All interviewed PSRs gave negative answers regarding the use of this tool. ANOVA determined a significant influence of the factor of the category of respondents on the variation of the prevalence of all tools of this technique (*p* ˂ 0.001). The Scheffé test pointed to significant differences (*p* ˂ 0.001) between the groups pharmacy consumers/pharmacists and pharmacy consumers/PSRs for all tools, except for free samples. 

**Figure 4:**
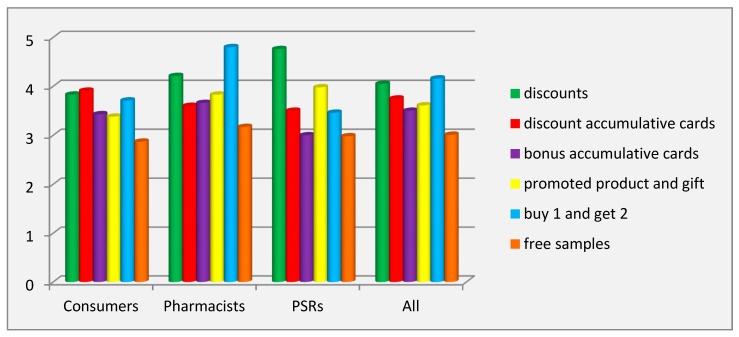
Effectiveness of the tools for the consumer promotion technique. Abbreviations: PSRs (pharmaceutical sales representatives).

The study of the effectiveness of consumer promotion tools revealed that, according to all respondents, the marketing tool buy 1 and get 2 (4.16 ± 1.71 points) had the greatest impact on the consumers’ purchasing decision, and the least effective tool was free samples of the promoted product (3.01 ± 1.77 points). Free samples of the promoted product ranked last in all groups of respondents in terms of effectiveness. PSRs put discounts (4.76 ± 1.67 points) in first place with a big difference from other tools, while pharmacists considered that the marketing tool buy 1 and get 2 (4.80 ± 1.53 points) had the strongest impact on the consumers’ purchasing decisions. According to the consumers’ answers, two tools of this technique were distinguished to have greater effectiveness: discount accumulation programs (3.91 ± 1.38 points) and discounts (3.83 ± 1.87 points) (Figure 4).

The values of the coefficient of variation showed significant inter-individual differences in all groups of respondents for all tools. The factor of the category of respondents had a significant influence (*p* < 0.001) on the variation of the evaluation of all tools of this technique, except for free samples, as highlighted by ANOVA. 

### 3.3. Trade Promotion (Motivating Pharmacists’ Trading Activity)

**Figure 5:**
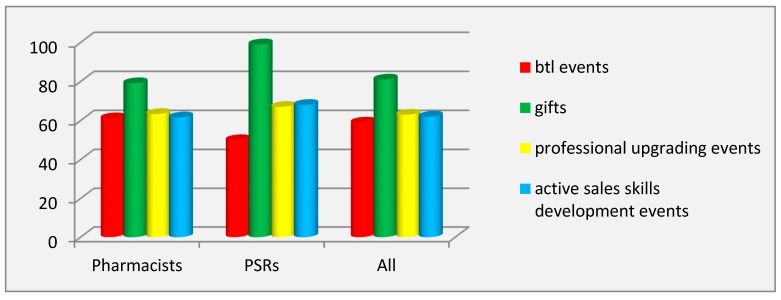
Prevalence of the tools of the trade promotion technique. Abbreviations: btl (below the line); PSRs (pharmaceutical sales representatives).

In the case of trade promotion techniques, only the opinions of PSRs and community pharmacists were studied because pharmacy consumers did not have to implement this technique in practice. The opinions of the respondents in the group of PSRs (RIP = 71%) and pharmacists (RIP = 66.32%) fully coincided: this technique ranked first in prevalence. It was found that the most prevalent tool within this technique was gifts when a certain level of retail sales (or wholesale purchases) of the promoted product was reached (RIP = 99% in the group of PSRs and 79.12% in the group of pharmacists). Btl events designed for motivating the pharmacist to dispense the promoted product (RIP in PSRs group = 50%) were not employed by pharmaceutical companies at the retail pharmaceutical market of Riyadh (Figure 5).

The findings of the values of the coefficient of variation showed inter-individual differences in both groups of respondents for the tools of this technique, except for btl events and gifts in the group of PSRs. Inter-group differences (*p* ˂ 0.001) were found for two tools of this technique: btl events and gifts.

**Figure 6:**
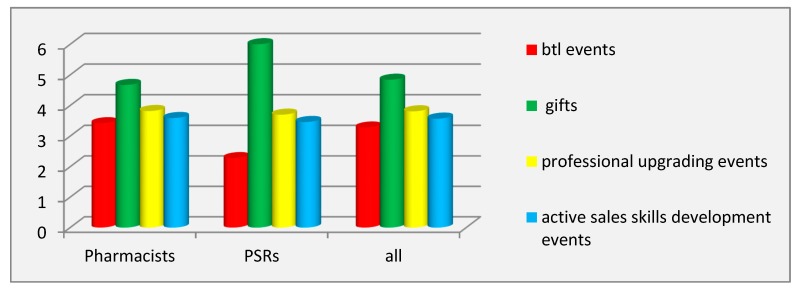
Effectiveness of the tools of the trade promotion technique. Abbreviations: btl (below the line); PSRs (pharmaceutical sales representatives).

Evaluation of the effectiveness of the tools for trade promotion showed a complete agreement of respondents of both groups (PSRs and pharmacists): gifts was named as the most effective tool. PSRs rated it with the highest possible score (6.00 ± 0.00), thereby identifying it as having the strongest motivational effect on pharmacists to dispense the promoted product. Btl events were considered to be the least effective tool for trade promotion (Figure 6).

The values of the coefficient of variation showed significant inter-individual differences in the evaluation of the effectiveness of all tools, except for gifts in the group of PSRs. The factor of the category of respondents had a moderate influence on the variation of the evaluation of the tools btl events and gifts, as pointed to by ANOVA.

## 4. Discussion

The findings of our research showed that consumers considered merchandising to be the most common marketing technique at community pharmacies. This could be explained by the fact that tools of merchandising are more apparent and obvious for consumers than tools of other promotional techniques. This fact is consistent with the essence of merchandising, which is to build effective marketing communications between a product and consumers. This finding matches those in the literature. For example, Dwight and Kulumbekova [15] noted merchandising as the main marketing tool most commonly employed at community pharmacies. At the same time, our research revealed that consumers considered that merchandising tools had the least impact on their purchasing decisions. Similarly, PSRs named merchandising as the least effective and the least prevalent sales promotion technique used at community pharmacies by pharmaceutical companies. This finding is confirmed by data available in the literature that tools of merchandising affect only 5.75% of consumers at a pharmacy [16].

Our research found that the most prevalent tool for consumer promotion was free samples of the promoted product. This result closely aligns with Zaki’s [17] conclusion that free product samples are the most accepted giveaways in the KSA and are considered to be the most suitable donation [17]. Similarly Al-Areefi et al. [18] claimed that in Yemen, free product samples were widely used alongside other gifts from pharmaceutical companies [18]. Notwithstanding the above, the findings of our research indicated that free samples of the promoted product were considered to be the least effective tool for consumer promotion by respondents of all groups. This means that free samples of the promoted product have little impact on the consumers’ purchasing decisions. 

According to our findings, the least prevalent tool for consumer promotion was discounts. This could be explained by the fact that fixed state prices are used at the retail level of the pharmaceutical market in the KSA. In fact, we found that the most effective tools for consumer promotion were price incentive tools. Therefore, pharmacy consumers and PSRs considered that discounts and discount accumulative programs had the strongest impact on consumers’ purchasing decisions, while pharmacists named the offer buy 1 and get 2 to be the most influential.

Our research showed the complete concurrence of PSRs and pharmacists’ opinions regarding the trade promotion tools: gifts when a certain level of retail sales (or wholesales purchases) of the promoted product is reached was named as the most effective and, at the same time, as the most frequently used. In other words, pharmaceutical companies mostly employed the tool which had the greatest motivational effect on pharmacists to dispense the promoted product, which in the end, strongly impacts on the consumers’ purchasing decisions. These findings match those in the literature. After all, more than half of pharmacy purchases are made as a result of direct or personal sales when a pharmacist plays a crucial role in a consumers’ purchasing decision [5,14,17,19,20].

However, despite its methodological strengths (ad hoc questionnaire, non-repeated random sampling technique, and representative sample), our study is not without limitations. The major shortcoming was, that given the exploratory nature of our investigation, we limited statistical analyses to a coefficient of variation, ANOVA, and post-hoc test without performing regression analyses or structural equation modeling, which would enable the understanding of the determinants of inter-individual and inter-group differences, make more robust causal inferences and build predictive models helpful to the stakeholders.

## 5. Conclusions

Most pharmaceutical companies invest time and money in the field of marketing and try to find the most effective promotion tools to increase their sales and revenue.

Previous research discussing pharmaceutical marketing in the KSA has failed to identify the most effective sales promotion techniques that had the strongest impact on consumers’ purchasing decisions.

Thus, this research fills a gap in knowledge in the existing literature by identifying the most prevalent sales promotion techniques used by pharmaceutical companies at the retail market of the KSA and by determining the most effective among them, that is to say, those that had the strongest impact on consumers’ purchasing decision according to the opinions of the different participants involved in the promotion process: PSRs, community pharmacists, and pharmacy consumers.

The study findings indicated that, according to PSRs and community pharmacists, the most effective and, at the same time, the most prevalent technique was trade promotion. Consumers named merchandising as the most common technique, but at the same time, they considered that the tools of consumer promotion technique had the strongest impact on their purchasing decisions.

The research findings identified that, according to all respondents, the following sales promotion tools had the strongest impact on consumers’ purchasing decisions: arrangement and design of showcases among the studied tools for merchandising; buy 1 and get 2, and discounts among the studied tools for consumer promotion; and gifts among the trade promotion studied tools. The findings showed that the same tools were named as the most common by all respondents. The tool free sample of promoted products appeared to be the most prevalent, but, at the same time, was the least effective.

Undertaking this research was of paramount significance not only because it fills important gaps in the existing scholarly literature, but it also offers pharmaceutical companies a better understanding of which sales promotion techniques have the strongest impact on consumers’ purchasing decisions, thereby helping companies focus on the most effective marketing methods to boost their sales revenue as well as to reduce their marketing expenses. This could lower the product costs passed on to consumers. At the same time, our findings could be useful to healthcare decision- and policy-makers in the process of developing the necessary policies for regulating pharmaceutical promotion in the KSA.

## Figures and Tables

**Table 1:** Socio-demographic characteristics: gender, age, job experience, level of education.

Respondents	N	Gender		Age				Job Experience				Level of Education			
		M	F	˂30	30–40	41–60	>60	˂1	4–1	10–5	>10	Bachelor	Master	Doctor	Other
Pharmacist	340	340	-	-	-	-	-	27	172	75	66	340	0	-	-
PSRs	50	34	16	-	-	-	-	4	22	24	0	49	1	-	-
Consumers	400	314	86	88	108	144	60	-	-	-	-	152	94	37	117
All	790	688	102	-	-	-	-	-	-	-	-	541	95	37	117

**Table 2:** Relative importance of prevalence and ranking of sales promotion techniques.

	PSRs		Pharmacists		Consumers	
Marketing Techniques	Relative Importance (%)	Rank	Relative Importance (%)	Rank	Relative Importance (%)	Rank
Consumer promotion	66.66	2	63.82	3	75.65	2
Merchandising	66.33	3	66.1	2	76.17	1
Trade promotion	71	1	66.32	1	-	-

**Table 3:** The mean evaluation of effectiveness and ranking of sales promotion techniques.

	PSRs		Pharmacists		Consumers	
Marketing Techniques	Mean Evaluation of Effectiveness	Rank	Mean Evaluation of Effectiveness	Rank	Mean Evaluation of Effectiveness	Rank
Consumer promotion	3.61	2	3.88	2	3.52	1
Merchandising	3.43	3	3.87	3	3.47	2
Trade promotion	3.86	1	3.9	1	-	-
